# Tissue-Specific Tolerance to High-Temperature and Nutrient-Poor Conditions in a Canopy-Forming Macroalga, Surviving at an Ocean Warming Hotspot

**DOI:** 10.3390/plants13121689

**Published:** 2024-06-18

**Authors:** Hikaru Endo, Masafumi Kodama, Ryoya Kawashima, Momochika Kumagai, Midori Matsuoka, Keigo Ebata, Suguru Okunishi

**Affiliations:** 1 Faculty of Fisheries, Kagoshima University, Kagoshima 890-0056, Japan; mkodama@fish.kagoshima-u.ac.jp (M.K.); k1693095kadai@gmail.com (R.K.); kumagai@fish.kagoshima-u.ac.jp (M.K.); m-matsuoka@fish.kagoshima-u.ac.jp (M.M.); ebata@fish.kagoshima-u.ac.jp (K.E.); okunishi@fish.kagoshima-u.ac.jp (S.O.); 2 United Graduate School of Agricultural Sciences, Kagoshima University, Kagoshima 890-0065, Japan

**Keywords:** climate change, tropicalization, heat stress, vegetative reproduction, marine macroalgal forest, *Sargassum*

## Abstract

Most canopy-forming macroalgae have disappeared from temperate reefs in southern Japan, one of the ocean warming hotspots, but *Sargassum nipponicum* is surviving in this region. As this species’ annual shoots emerge from holdfasts during summer, both plant components may be highly tolerant to warm and nutrient-poor conditions in this season. The present study examined the effects of temperature and nutrient conditions on holdfast growth, shoot emergence from holdfasts, and shoot growth in *S*. *nipponicum* samples collected in Tanegashima Island, southern Japan. The summer temperature in this region (30 °C) allowed holdfast growth and shoot emergence but inhibited shoot growth. Nutrient-poor conditions had limited effects on the first two parameters but suppressed shoot growth. These results suggested that during warm summers and under nutrient-poor conditions in southern Japan, shoots can emerge from *S. nipponicum* holdfasts but cannot further grow. Additionally, nutrient loading from a nearby river was higher at the only site dominated by *S. nipponicum,* than at the other sites where this species was absent on Tanegashima Island. This was observed especially between autumn and winter, implying that such a nutrient-rich environment may contribute to shoot growth in *S*. *nipponicum* and to the persistence of its population in the area.

## 1. Introduction

Marine macroalgal forests dominated by canopy-forming large brown algae, such as kelp (Laminariales) and fucoid (Fucales) species, have ecologically and economically important roles, including the removal of carbon and nutrients from seawater, and the provision of food, habitats, and spawning grounds for various marine organisms in temperate reef ecosystems [1,2,3,4].

These temperate macroalgae are basically adapted to temperate reef environments, which are characterized by relatively warm temperatures and nutrient-poor conditions during summer, and by cold temperatures and nutrient-rich conditions during winter. For example, perennial kelp species survive during the nutrient-poor summer by using the internal nitrogen reserves accumulated during the nutrient-rich winter [5,6]. Annual kelp species grow between autumn and spring in the form of macroscopic sporophytes and survive during the warm summer as microscopic gametophytes, which tolerate higher temperatures compared to sporophytes [7,8]. Some perennial fucoid algae, belonging to the genus *Sargassum*, lose their annual shoots before summer and survive during this season in the form of small bodies with newly emerged short shoots, stipes (analogous to stems), and holdfasts (analogous to rhizoids) [9,10]; which can tolerate high temperatures [11].

However, above-average temperatures under nutrient-poor conditions during summer decrease the growth and survival rates of these canopy-forming macroalgae [12,13,14,15,16,17]. Therefore, as a result of recent ocean warming, marine forests in temperate reefs have experienced a decline at the regional level [18]. Moreover, ocean warming has also been shown to intensify macroalgal herbivory by fish in tropicalized temperate waters [19]. At the same time, phase shifts from coral reefs to states dominated by macroalgae, such as *Sargassum* species, have been reported in tropical waters, specifically at sites where the nutrient loading from nearby rivers was high, even if the herbivorous fish were abundant [20]. This implies that kelp and fucoid species growing in tropicalized temperate waters may also survive at sites with a high nutrient loading from nearby rivers, even under intensive fish herbivory.

The coastal water around southern Japan is one of the ocean warming hotspots [19]. Almost all kelp and fucoid species found in this region have shifted their distributional range toward the pole between 1970 and 2000 [21]. However, such a shift has not been observed in several species, including the two fucoid algae *Sargassum fusiforme* (Harvey) Setchell and *S. nipponicum* Yendo [21]. Both of these macroalgae have filamentous holdfasts [22], from which annual shoots emerge during summer [10,23]. The shoots grow between autumn and spring and decay during the following summer after the production of propagules [10,23]. Previous studies have shown that the upper limit temperature for the survival of both *S. fusiforme* propagules and shoots in nutrient-enriched seawater was 32 °C [24,25], which is higher than the summer seawater temperature of ca. 30 °C recorded in the Kagoshima and Okinawa Prefectures, in southern Japan [11,26]. A more recent study reported that *S. fusiforme* propagules exhibited positive growth rates at 30 °C in nutrient-enriched seawater but negative values at the same temperature in nutrient-poor natural seawater, while its holdfasts without shoots showed positive growth rates even at 32 °C in natural seawater [11]. Additionally, the negative effect of elevated summer temperatures on the growth rate of *S. fusiforme* shoots has been shown to be antagonized by decreased irradiance [27]. However, no study has comprehensively evaluated the effects of temperature, irradiance, and nutrient conditions on growth (including holdfast growth, shoot emergence from holdfasts, and shoot growth) during summer in either *S.fusiforme* or *S. nipponicum*.

*S. nipponicum* generally grows at shallow depths along wave-exposed coasts in the warm-temperate and subtropical waters around southern Japan [22]. This species has previously been observed along the northern coasts of Tanegashima Island, southern Japan, but has now almost disappeared [28]. However, a marine forest dominated by *S. nipponicum* was found at a single site along this coast in our preliminary survey (site A in Figure S1). Hence, the present study examined (1) the combined effects of temperature (20–30 °C) and irradiance, and (2) the effect of high temperature (30–38 °C) on holdfast growth, shoot emergence from holdfasts, and shoot growth in this algal species by conducting two laboratory culture experiments. Moreover, the effect of nutrient concentration on these variables was also evaluated. The temperature and nutrient conditions tested in the experiments were decided based on field survey data on seasonal changes in seawater temperature, *S. nipponicum* shoot length, as well as dissolved inorganic nitrogen (DIN) and dissolved inorganic phosphorus (DIP) concentrations recorded in seawater and river water along the northern coast of Tanegashima Island.

## 2. Results

### 2.1. Seasonal Changes in Seawater Temperature and Shoot Length

**Figure 1:**
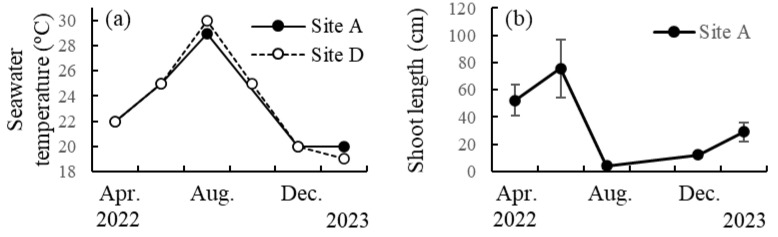
(**a**) Seasonal changes in seawater temperature at two sites A and D in Tanegashima Island. (**b**) Seasonal change in shoot length of *Sargassum nipponicum* at site A. Values are expressed as means ± standard deviations.

At two sites along the Tanegashima coast (sites A and D in Figure S1), seawater temperature increased from 22 °C in April 2022 to 29–30 °C in August (i.e., summer) and decreased to 19–20 °C in February 2023 (i.e., winter) (Figure 1). The mean shoot length of *S. nipponicum* at site A (Figure S1) gradually increased from 52.2 cm in April 2022 to 75.7 cm in June and rapidly decreased to 4.1 cm in August (i.e., summer). Then, it gradually increased to 12.3 cm in December (i.e., autumn) and rapidly reached 29.0 cm in February 2023 (i.e., winter) (Figure 1).

### 2.2. Combined Effects of Temperature and Irradiance

**Figure 2:**
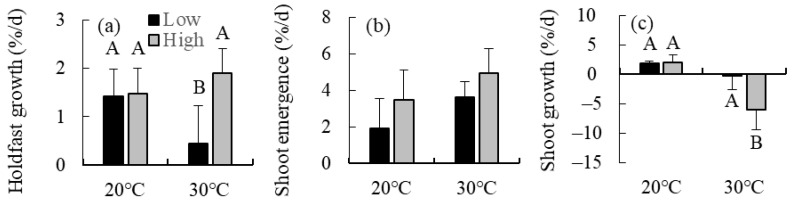
(**a**) Holdfast grow rates, (**b**) shoot emergence rate, and (**c**) shoot growth rate of the brown alga *Sargassum nipponicum* cultured in two temperature levels (20 and 30 °C) combined with two irradiance levels (low: 30 µmol photons m^−2^ s^−1^ and high: 130 µmol photons m^−2^ s^−1^). Values are expressed as means ± standard deviations. Different uppercase letters indicate statistical significance (*p* < 0.05) among the different treatments.

The holdfast growth rate was significantly affected by irradiance, and a significant interaction between irradiance and temperature was also shown to influence this variable (Table 1), indicating that the effect of irradiance varied in response to temperature changes. In fact, the holdfast growth rate declined in response to irradiance decreasing from 130 to 30 µmol photons m^−2^ s^−1^ at 30 °C, while no significant difference was detected between the two irradiance treatments at 20 °C (Figure 2).

The rate of shoot emergence from holdfasts was significantly affected by temperature and irradiance (Table 1), increasing as these two parameters increased from 20 °C to 30 °C and from 30 to 130 µmol photons m^−2^ s^−1^, respectively (Figure 2).

Temperature and irradiance significantly affected the shoot growth rate as well, and the significant interaction between them was also shown to exert an effect on this variable (Table 1). The shoot growth rate decreased in response to irradiance increasing from 30 to 130 µmol photons m^−2^ s^−1^ at 30 °C, whereas no significant differences were detected between the two irradiance treatments at 20 °C (Figure 2). Parallelly, the growth rate decreased in response to temperatures increasing from 20 °C to 30 °C at 130 µmol photons m^−2^ s^−1^, whereas no significant difference was observed between the two temperature treatments at 30 µmol photons m^−2^ s^−1^.

### 2.3. Effect of High Temperature

**Figure 3:**
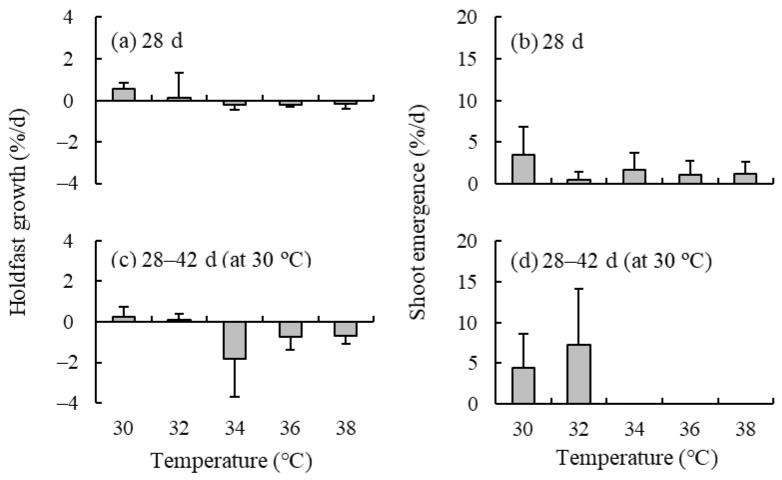
(**a**) Holdfast growth rates and (**b**) shoot emergence rate of the brown alga *Sargassum nipponicum* cultured in five temperature levels (30, 32, 34, 36, and 38 °C) for 28 d. (**c**,**d**) These variables after following 14 d culture at 30 °C for all the treatments. Values are expressed as means ± standard deviations.

The mean holdfast growth rates during 28 d of culture in natural seawater were positive at 30 °C and 32 °C but negative at 34, 36, and 38 °C (Figure 3). The emergence of shoots from holdfasts was observed at all tested temperatures during this period. After 14 d of culture at 30 °C, the holdfast growth rates and shoot emergence rate in all treatments were positive at 30 and 32 °C but negative at 34, 36, and 38 °C. In contrast, the shoot growth rates were negative (values ranging from −8.87 to −0.84% d^−1^) at all the above-mentioned temperatures (30–38 °C) after only 7 d of culture (Figure S1). The appearance of the holdfast did not change even at high temperatures, whereas the shoots were apparently decomposed.

### 2.4. Seasonal Changes in DIN and DIP Concentrations

**Figure 4:**
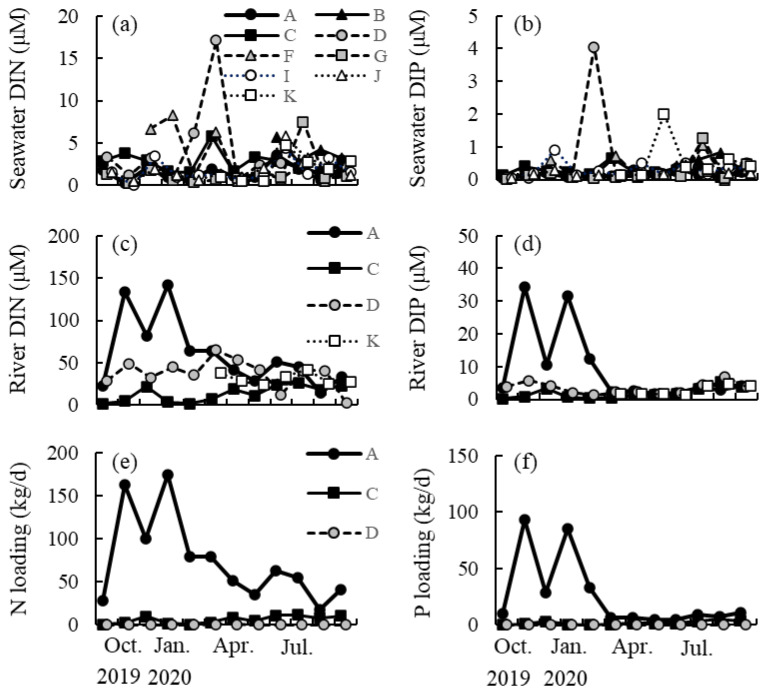
Seasonal changes in (**a**) dissolved inorganic nitrogen (DIN) and (**b**) dissolved inorganic phosphorus (DIP) concentrations of seawater collected at 9 sites (A, B, C, D, F, G, I, J, and K in Figure S1) in Tanegashima Island. Seasonal changes in (**c**) DIN and (**d**) DIP concentrations of river water collected at 4 sites (A, C, D, and K). Seasonal changes in (**e**) nitrogen and (**f**) phosphorus loading transported by the river toward the coast at 3 sites (A, C, and D).

The seawater DIN and DIP concentrations, which were recorded seasonally between October 2019 and September 2020 from a depth of 2 m at nine different sites along the northern Tanegashima coast, ranged from 0.31 to 17.07 μM and from 0.02 to 4.05 μM, respectively (pooled data) (Figure 4). Values did not tend to be higher at site A, where *S. nipponicum* was predominant on the reef, than at the other sites where this species was absent. The mean DIN/DIP ratio was 12.8.

The river water DIN and DIP concentrations, which were recorded seasonally between October 2019 and September 2020 from the surface layer at four sites in the river mouth along the northern Tanegashima coast, ranged from 1.95 to 142.23 μM and from 0.20 to 34.20 μM, respectively (pooled data). These values tended to be higher at site A (DIN: 64.5–142.2 μM, DIP: 10.4–34.2 μM) between November 2019 and February 2020 (i.e., between autumn and winter) than at the other sites (DIN: 2.0–49.4 μM, DIP: 0.4–5.7 μM), although such a tendency was not observed between March and September 2020 (i.e., between spring and summer). The mean DIN/DIP ratio was 12.0. The nitrogen and phosphorus loading transported by the river toward the coast was estimated by multiplying the DIN and DIP values by the river flow rate and tended to be higher at site A (Nitrogen: 18.12–174.97 kg d^−1^, Phosphorus: 4.89–92.99 kg d^−1^) than at sites C (Nitrogen: 0.88–11.98 kg d^−1^, Phosphorus: 0.20–5.28 kg d^−1^) and D (Nitrogen: 0.03–0.74 kg d^−1^, Phosphorus: 0.03–0.18 kg d^−1^).

### 2.5. Effect of Nutrient Concentration

**Figure 5:**
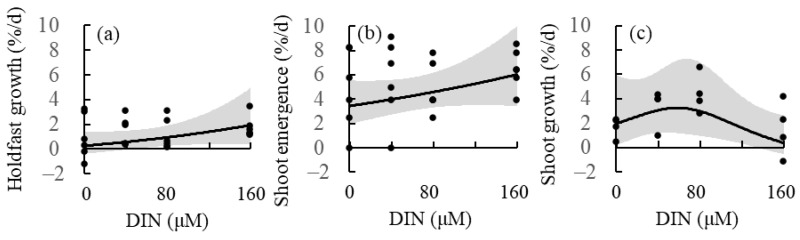
(**a**) Holdfast grow rate, (**b**) shoot emergence rate, and (**c**) shoot growth rate of the brown alga *Sargassum nipponicum* cultured in four nutrient levels (DIN/DIP = 0/0, 40/4, 80/8, and 160/16 μM). Solid lines and gray zones indicate mean and confidence intervals, respectively, estimated using linear (**a**,**b**) and generalized additive (**c**) models.

A null model was compared with a linear model to explore the relationships between nutrient concentration and *S. nipponicum* holdfast growth and shoot emergence rates using *F* test (Figure 5). The results revealed an insignificant effect of nutrient concentration on these variables (*p* = 0.153 and 0.196, respectively). In contrast, based on the results of a generalized additive model, the shoot growth rate increased from 1.95% d^−1^ at DIN/DIP = 0/0 μM to a peak of 3.25% d^−1^ at DIN/DIP = 59/5.9 μM, and finally decreased to 0.39% d^−1^ at DIN/DIP = 160/16 μM.

## 3. Discussion

Charan et al. [27] reported that the negative effect on shoot growth in *S. fusiforme* caused by summer temperature increasing from 23 to 26 °C was synergized by the co-occurring increase in irradiance from 30 to 180 µmol photons m^−2^ s^−1^, probably because excess light energy under warm temperature caused photoinhibition of this species [29]. Similarly, in the present study, the negative effect on shoot growth in *S. nipponicum* caused by temperature increasing from 20 to 30 °C was synergized by irradiance increasing from 30 to 130 µmol photons m^−2^ s^−1^, although the mean growth rates were negative at 30 °C even at the lowest irradiance level. In contrast, the negative effect of temperature elevation on holdfast growth was antagonized by the increased irradiance (from 30 to 130 µmol photons m^−2^ s^−1^). This may be due to the high respiration rates exceeding the rate of photosynthesis under warm conditions [30]. Thus, the response to the simultaneous increase in temperature and irradiance differed between algal tissues even within the same species. At the same time, the rate of shoot emergence from *S. nipponicum* holdfasts was shown to increase in response to the increasing temperature and irradiance. These results indicated that *S. nipponicum* holdfasts can grow and shoots can emerge from them in the summer (at 30 °C), but these shoots cannot further grow at this temperature, especially under strong irradiance at shallow depths.

The *Sargassum* species distributed along the Japanese coasts are classified into temperate and subtropical (warm temperate) based on their distributional range [31]. The upper limit temperature for shoot survival in temperate *Sargassum* species, including *S. fusiforme*, under nutrient-enriched conditions has been reported to range from 27 to 32 °C [25,32]. However, shoot survival under these temperatures has rarely been evaluated in subtropical *Sargassum* species, including *S. nipponicum*. In the present study, *S. nipponicum* shoots showed negative growth rates at 30–38 °C in nutrient-poor natural seawater after only 7 d of culture. In contrast, holdfasts exhibited positive growth rates at 30 and 32 °C during the 28 d culture experiment. Moreover, shoot emergence from holdfasts was accelerated by a decrease in temperature from 32 to 30 °C. These results suggested that the strong tolerance of holdfasts to high temperatures ensured the survival of *S. nipponicum* during the warm summer in southern Japan, as reported for *S. fusiforme* [11].

Schaffelke and Klumpp [33] reported that shoot growth in *Sargassum baccularia* during 28 d of culture in nutrient-poor natural seawater was enhanced by a preliminary pulsed nutrient supply with DIN/DIP ratios of 5/0.5–50/5 μM for 24 h compared to the non-supplied control. During the 14 d culture experiment in the present study, the *S. nipponicum* shoot growth rates were optimal when DIN and DIP were supplied at a ratio of 59/5.9 μM once every 3–4 d, and decreased in response to further increases and decreases in nutrient concentrations. In contrast, the effect of nutrient concentration on holdfast growth and shoot emergence rates were relatively small. These results suggested that *S. nipponicum* holdfasts can grow and shoots can emerge from them under both nutrient-poor and nutrient-rich conditions, but these shoots cannot further develop unless nutrient concentrations increase in the growing environment.

In perennial *Sargassum* species growing in temperate Japan, annual shoots generally emerge from the holdfasts or stipes during summer, grow rapidly between autumn and spring, and decay during the following summer after the production of propagules [9,10]. In contrast, the shoots of *Sargassum* species distributed in subtropical Japan often do not grow between summer and autumn, but grow rapidly from winter to spring [23,34,35,36], as observed in the present study. This delay has been speculated to be due to fish herbivory [34,35,36], which was reported to be more intensive between summer and autumn than during winter in this region [37]. However, in the present study, it was shown that shoot growth in the subtropical species *S. nipponicum* was strongly inhibited by high temperature, i.e., 30 °C, which is commonly recorded during summer in southern Japan [11,26]. Moreover, shoot growth was also suppressed by decreased nutrient concentration. Hence, the delayed *S. nipponicum* shoot growth observed in the study area may be caused not only by fish herbivory but also by warm temperatures and low nutrient conditions during summer.

Yoshida and Shimabukuro [10] showed that the filamentous holdfasts of *S. fusiforme* decayed during autumn after shoot emergence during summer but were regenerated from growing shoots during winter. The same was reported in Takase and Tanaka [23] for *S. nipponicum*. Thus, in these species, shoot growth between autumn and winter is necessary for holdfast regeneration and therefore for population persistence. However, the present study showed that shoot growth in *S. nipponicum* was suppressed by both high temperature and nutrient-poor conditions. These findings suggested that a temperature decline and an increase in nutrient concentration between summer and autumn were required to ensure shoot growth in this species and the persistence of its population.

Adams et al. [20] reported the occurrence of phase shifts from coral reefs to states dominated by macroalgae, such as *Sargassum* spp., at sites on a tropical island, where the nutrient loading from nearby rivers was high. In the present study, the seawater DIN and DIP concentrations did not tend to be higher at site A, which was the only site where *S. nipponicum* was predominant on the reef, than at the other sites where this species was absent along the northern coast of Tanegashima Island. In contrast, the DIN and DIP concentrations in river waters were higher at site A than at the other sites between autumn and winter. Shoot growth in *S. nipponicum* was optimal at DIN/DIP concentrations of 59/5.9 μM, which is more similar to the nutrient content of river waters than to that of seawater in Tanegashima Island. Moreover, the nutrient loading transported from the river toward the coast at site A was obviously higher than that at the other sites throughout the year. Therefore, such high nutrient concentrations supplied by river waters may allow shoot growth in *S. nipponicum* and the persistence of its population at this site. However, nitrogen stable isotope analysis of the algae and waters [38] is necessary to confirm whether the nutrients supplied by the river are being assimilated by *S. nipponicum*.

The present study revealed that, in *S. nipponicum*, holdfasts were more tolerant to high temperature and nutrient-poor conditions than the annual shoots. The strong tolerance of the holdfasts ensured survival under warm and nutrient-poor conditions during summer in southern Japan. However, a decrease in temperature and an increase in nutrient concentrations between summer and autumn were required for the shoots to grow. Shoot growth in *S. nipponicum* was enhanced at a DIN/DIP concentration of 59/5.9 μM, which was similar to that observed in river water, but was suppressed at the concentrations of seawater, observed in seawater at Tanegashima Island. Therefore, marine forests dominated by *S. nipponicum* may persist in southern Japan under ocean warming, especially at the site where nutrient loading from the nearby river is high.

## 4. Materials and Methods

### 4.1. Seasonal Changes in Seawater Temperature and Shoot Length

Seawater temperatures at sites A and D (Figure S1) in Tanegashima Island were recorded at a depth of 2 m using a diving computer during diving surveys in April, June, August, October, and December 2022 and in February 2023, although site A was not surveyed in October 2022 due to strong wave action. Seven *S. nipponicum* individuals were randomly collected from a depth of 1–2 m at site A during the surveys, and their shoot length was measured using a ruler immediately after collection.

### 4.2. Combined Effects of Temperature and Irradiance

Six *S. nipponicum* individuals with relatively large holdfasts were collected in July 2019 from site A (Figure S1). A total of 24 holdfast segments measuring 5 mm in length were cut from the specimens. The wet weight of each segment (initial value) was measured using an electronic balance (with an accuracy of 0.1 mg) after removing excess moisture by blotting on paper towels. These segments were randomized into four groups of six specimens with a similar size distribution. Each group was subjected to one of four different treatments consisting of two temperature levels (20 and 30 °C) and two irradiance levels (30 and 130 µmol photons m^−2^ s^−1^).

The two temperature levels were established based on the temperature range recorded at the site of collection (ca. 20 °C during winter and ca. 30 °C during summer). The temperature of ca. 30 °C is commonly recorded during summer in the Kagoshima and Okinawa Prefectures, southern Japan [11,26]. As no information was available on the optimal irradiance for *S. nipponicum* growth, the levels of this parameter were established based on data reported for the related species *S. fusiforme*, which grows at similar shallow depths [22]. In this species, the holdfast growth rate was shown to increase in response to irradiance increasing from 30 to 130 µmol photons m^−2^ s^−1^ but not under further increases in irradiance from 130 to 300 µmol photons m^−2^ s^−1^ [39]. Irradiance was measured using a light meter (LI-250A, LI-COR, Lincoln, NE, USA) with a spherical quantum sensor (LI-193A, LI-COR, Lincoln, NE, USA)

The *S. nipponicum* specimens were placed in a Petri dish (one segment per dish) containing 30 mL of sterile natural seawater and were cultured for 28 d under a 12 h L: 12 h D photoperiod. The sterile seawater in each dish was changed every 7 d. At the end of the experiment, the wet weight and number of new shoots emerged from the cultured segments (final value) were determined. The relative holdfast growth rate (% d^−1^) and shoot emergence rate were calculated as 100 × ln (final value/initial value)/culture d.

Moreover, four *S. nipponicum* individuals with more than four short shoots were collected in November 2019 from site A. A total of 16 shoots were cut from these specimens, and their initial wet weight was measured. Each shoot was then placed in a flask (one per flask) containing 300 mL of sterile seawater. Subsequently, these shoots were incubated under four different temperature and irradiance conditions (four shoots per treatment). Sterile seawater was used as culture medium. Incubation was stopped after 7 d because some of the shoots cultured at 30 °C started withering. The final wet weight of the shoots was determined, and their relative growth rate was calculated.

The combined effects of temperature and irradiance on the holdfast growth rate, rate of shoot emergence from holdfasts, and shoot growth rate were tested using two-way ANOVA and Tukey’s multiple comparison tests. These variables were logarithmically transformed in some cases to ensure the assumptions of normality and variance homogeneity in the data. All statistical analyses in the present study were performed in R version 4.1.1 (R Development Core Team, Vienna, Austria).

### 4.3. Effect of High Temperature

Based on the procedures described above, six *S. nipponicum* individuals were collected in July 2020 from site A. A total of 30 holdfast segments were cut from them and their initial wet weight was measured. These shoots were cultured for 28 d at five different temperatures (i.e., 30, 32, 34, 36, and 38 °C) and under an irradiance of 130 µmol photons m^−2^ s^−1^ and a 12 h L: 12 h D photoperiod. Sterile natural seawater was used as culture medium and was changed every 7 d. The final wet weight and number of new shoots that emerged from cultured segments were determined. Then, the relative holdfast growth rate (% d^−1^) and shoot emergence rate were calculated.

Four *S. nipponicum* individuals were collected in November 2020 from site A. A total of 16 shoots were cut from them and their wet weight was measured. These shoots were also cultured at the five different temperatures mentioned above. Sterile seawater was used as culture medium. This culture was stopped after 7 d because almost all shoots withered. The final wet weight of the shoots was determined, and the relative shoot growth rate was calculated.

The effect of high temperature was evaluated based on whether the mean growth rates were positive or negative and whether the shoot emergence rate was zero or higher, instead of using ANOVA, which requires homogeneity of the data.

### 4.4. Seasonal Changes in DIN and DIP Concentrations

A total of 15 mL of seawater was sampled from a depth of 2 m using a van Dorn water sampler at nine sites along the northern coast of Tanegashima Island between October 2019 and September 2020 (Figure S1). Moreover, river surface water was sampled from four sites at the river mouth using a 15 mL bottle during the same period. These water samples were filtered immediately after collection and transported to the laboratory in a cooler box, where they were frozen at −30 °C. Their DIN and DIP concentrations were measured using a continuous flow analyzer (SWAAT, BL TEC K.K., Tokyo, Japan). Additionally, water depth at the river mouth was measured using a surveying rod every 30 or 100 cm, depending on the river width, in the direction of crossing at the three sites (A, C, and D), and the cross-sectional area of the river mouth was calculated. The time required for the plastic bottle to flow over a distance of 10 m through the center of the river was measured five times to determine flow velocity. Then, the flow rate was calculated by multiplying the flow velocity by the cross-sectional area. The nitrogen and phosphorus loading transported from the river toward the coast were estimated by multiplying the flow rate by the DIN and DIP concentrations, respectively.

### 4.5. Effects of Nutrient Concentration

A total of 24 holdfast segments were cut from the six *S. nipponicum* individuals collected in July 2020. These segments were weighed and cultured for 28 d under four different nutrient conditions and exposure to 130 µmol photons m^−2^ s^−1^ with a 12 h L: 12 h D photoperiod. The four tested DIN concentrations were 0, 40, 80, and 160 μM, while the tested DIP concentrations were 0, 4, 8, and 16 μM. These nutrient conditions were created by adding NaNO_3_ and Na_2_HPO_4_ solutions to artificial seawater without nitrogen or phosphorus (Marine art SF-1, Tomita Pharmaceutical Co., Ltd., Naruto, Japan). The culture media were changed every 7 d. The final wet weight and number of new shoots emerged from the cultured segments were determined. Then, the relative holdfast growth rate (% d^−1^) and shoot emergence rate were calculated.

A total of 16 shoots were cut from the 4 *S. nipponicum* individuals collected in November 2020. These shoots were weighed and cultured under the four different nutrient conditions as described above. Then, the final wet weight of the shoots was determined, and the relative shoot growth rate was calculated.

Because the holdfast growth and shoot emergence rates tended to increase with increasing nutrient concentrations, linear models were applied to determine the relationship between nutrient concentration and these variables used the *lm* function in R. The relationship between the fitted values and model residuals was visually checked using the ‘*autoplot*’ function in the ‘*ggfortify*’ package. The differences between the null model and the model of interest were tested using the *F* test. As the shoot growth rate increased peaked at nutrient concentrations between 0 and 160 μM, a generalized additive model was applied to explore the relationship between nutrient concentration and this variable and estimate the optimal nutrient concentration and peak shoot growth rate using the *gam* function in the ‘*mgcv*’ package. The relationship between the fitted value and the model response was visually checked using the ‘*gam.check*’ function. The differences between the linear and generalized additive models were tested using the *F* test. These variables were logarithmically transformed in some cases, and the goodness-of-fit of the model was assessed based on the Akaike information criterion.

## Figures and Tables

**Table 1:** ANOVA results on the effects of temperature (T) and irradiance (I) on variables.

Variables	Factor	df	SS	MS	*F*	*p*	
Holdfast growth rate	T	1	0.487	0.487	1.345	0.260	
	I	1	3.399	3.399	9.387	0.006	*
	T * I	1	2.977	2.977	8.223	0.010	*
Shoot emergence rate	T	1	15.39	15.386	7.771	0.011	*
	I	1	12.34	12.342	6.234	0.021	*
	T * I	1	0.090	0.088	0.045	0.835	
Shoot growth rate	T	1	104.47	104.470	22.847	<0.001	*
	I	1	30.040	30.040	6.570	0.025	*
	T * I	1	35.560	35.560	7.776	0.016	*

* Statistical significance.

## Data Availability

The original contributions presented in the study are included in the article/Supplementary Materials, further inquiries can be directed to the corresponding author/s.

## Supplementary Materials

The following supporting information can be downloaded at https://www.mdpi.com/article/10.3390/plants13121689/s1, Figure S1: Maps showing (a) Japan, (b) Kyusyu Island, and (c) northern part of Tanegashima Island, Kagoshima Prefecture, southern Japan. Filled and open circles indicate the sites where *Sargassum nipponicum* was observed and those where *Sargassum* species was not found, respectively, in our surveys conducted between July 2019 and July 2021. *S. nipponicum* dominated at 1–2 m depth on the reef at site A but was found only in a very limited area at site B. Open squares indicate the sites of seawater collection between October 2019 and September 2020. This file includes the minimal dataset of this study.
